# Single Nucleotide Polymorphisms in Taste Receptor Genes Are Associated with Snacking Patterns of Preschool-Aged Children in the Guelph Family Health Study: A Pilot Study

**DOI:** 10.3390/nu10020153

**Published:** 2018-01-30

**Authors:** Elie Chamoun, Joy M. Hutchinson, Owen Krystia, Julia A. Mirotta, David M. Mutch, Andrea C. Buchholz, Alison M. Duncan, Gerarda Darlington, Jess Haines, David W. L. Ma, Guelph Family Health Study

**Affiliations:** 1 Department of Human Health and Nutritional Sciences, University of Guelph, Guelph, ON N1G 2W1, Canada; echamoun@uoguelph.ca (E.C.); jmirotta@uoguelph.ca (J.A.M.); dmutch@uoguelph.ca (D.M.M.); amduncan@uoguelph.ca (A.M.D.); 2 Department of Family Relations and Applied Nutrition, University of Guelph, Guelph, ON N1G 2W1, Canada; mackayj@uoguelph.ca (J.M.H.); okrystia@uoguelph.ca (O.K.); abuchhol@uoguelph.ca (A.C.B.); jhaines@uoguelph.ca (J.H.); 3 Department of Mathematics and Statistics, University of Guelph, Guelph, ON N1G 2W1, Canada; gdarling@uoguelph.ca; 4 University of Guelph, Guelph, ON N1G 2W1, Canada; guelphfamilyhealthstudy@gmail.com

**Keywords:** genetics, taste, children, snacking

## Abstract

Snacking is an integral component of eating habits in young children that is often overlooked in nutrition research. While snacking is a substantial source of calories in preschoolers’ diets, there is limited knowledge about the factors that drive snacking patterns. The genetics of taste may help to better understand the snacking patterns of children. The rs1761667 single nucleotide polymorphism (SNP) in the *CD36* gene has been linked to fat taste sensitivity, the rs35874116 SNP in the *TAS1R2* gene has been related to sweet taste preference, and the rs713598 SNP in the *TAS2R38* gene has been associated with aversion to bitter, green leafy vegetables. This study seeks to determine the cross-sectional associations between three taste receptor SNPs and snacking patterns among preschoolers in the Guelph Family Health Study. Preschoolers’ snack quality, quantity, and frequency were assessed using three-day food records and saliva was collected for SNP genotyping (*n* = 47). Children with the TT genotype in *TAS1R2* consumed snacks with significantly more calories from sugar, and these snacks were consumed mostly in the evening. Total energy density of snacks was highest in the CC and CG genotypes compared to the GG genotype in *TAS2R38*, and also greater in the AA genotype in *CD36* compared to G allele carriers, however this difference was not individually attributable to energy from fat, carbohydrates, sugar, or protein. Genetic variation in taste receptors may influence snacking patterns of preschoolers.

## 1. Introduction

The prevalence of overweight and obesity in Canada has been growing at an alarming rate, and children are not exempt from this epidemic growth. According to the World Health Organization, 31.5% of children and adolescents aged 5–17 years old—an estimated 1.6 million Canadians—were classified as overweight (19.8%) or obese (11.7%) from 2009 to 2011 [1]. The accessibility of palatable, processed foods high in fats and sugars has increased in modern society. Chronic diseases such as obesity, cardiovascular disease (CVD), type 2 diabetes (T2D), and metabolic syndrome (MetS) have been partially attributed to poor eating habits in those compelled by the hedonic qualities of food [2]. The taste of food is a particularly important factor for parents when selecting foods for themselves and their children [3]. Through the individual selection of unhealthy foods, taste perception may therefore be an important determinant of chronic disease risk. 

Research on the genetic predisposition to selecting specific foods based on taste perception may be significant in advancing our fundamental knowledge of genetic factors influencing habitual dietary intake and the development of chronic disease. Genetic variation in taste perception can be characterized in terms of single nucleotide polymorphisms (SNPs) in taste receptor genes. Taste receptor function for sweet, fat, and bitter tastes forms much of the basis for the known inter-individual differences in taste perception [4,5]. The fat taste receptor cluster determinant 36 (CD36), the sweet taste receptor type 1 member 2 (T1R2) and sweet taste receptor type 1 member 3 (T1R3), and the bitter taste receptor T2R38 contain common polymorphisms that may alter taste perception, food preference, and consequently food selection [2].

Research related to fat taste perception has focused on the fatty acid translocase *CD36* gene. Decreases in fatty acid taste sensitivity have been associated with the AA genotype of the rs1761667 SNP in the *CD36* gene [6]. A lower oral sensitivity to fatty acids has been found to associate with a higher preference and consumption of dietary fat [7]. However, little is known about the effect of this SNP on dietary intake in children. 

The heterodimer T1R2 and T1R3 allows humans to taste a variety of sweet substances [8,9], and genetic variations in *TAS1R2* may pertain to changes in taste sensitivity to sugar [10,11,12,13,14,15,16,17,18,19]. Specifically, the TT genotype of the rs35874116 SNP in the *TAS1R2* gene has been linked to habitual sugar consumption in overweight and obese adults [20] and children [21]. In children, dental caries risk is a concern associated with the consumption of sugar [22]. The prevalence of dental caries was observed to be associated with the rs35874116 SNP [23]. Research to date has yet to show a relationship between the rs35874116 SNP and sugar consumption in children.

The perceptions of bitter and sweet tastes interact in such a way to affect eating habits, especially in children [4]. Consuming bitter compounds generally results in food rejection, an evolutionary adaptation to avoid toxic substances such as rancid fat, hydrolyzed protein and plant alkaloids [24,25]. Sensitivity to bitterness may lead to the avoidance of Brassica vegetables rich in fiber, thereby potentially being replaced with the consumption of energy-dense foods rich in sugar [26,27]. These eating habits have the potential to increase the risk of obesity, CVD, and cancer [28]. Kim et al. (2003) showed that the bitter receptor T2R38 mediated the sensitivity to PTC, a thiol compound chemically related to those found in green, leafy vegetables (Brassica vegetables) [29]. Three common SNPs in the *TAS2R38* gene result in amino acid substitutions at residues P49A (rs713598), A262V (rs1726866) and V296I (rs10246939). The C allele of the rs713598 SNP is associated with the PAV haplotype while the G allele is associated with the AVI haplotype. Individuals carrying two copies of the PAV haplotype typically have a strong aversion to the bitterness of PTC. Those who seldom taste PTC carry two copies of the AVI haplotype while heterozygotes (PAV/AVI) typically have an intermediate taste phenotype [30]. 

The prevalence of snacking, or the consumption of foods and/or beverages between meals, is increasing in children as 98% of U.S. children snacked daily in 2006 compared to 74% in 1977 [31]. Young children are especially inclined to snacking and typically consume small meals throughout the day rather than larger meals [32]. These snacks are frequently energy-dense and nutrient-poor choices such as desserts, salty snacks, and sugary drinks which lead to excess energy consumption [32]. While existing evidence identifies snacking as a substantial source of calories in preschoolers’ diets, there is limited knowledge about the factors that drive snacking patterns, including snacking frequency and the quantity and quality of snacks consumed. The genetics of taste is one factor that may help better understand snacking patterns of children. Studies examining the relationship between the genetics of taste and snacking in children are limited despite existing evidence that taste receptor SNPs may impact taste perception, and therefore food selection [2]. Examining snacking patterns among children may provide important insight into the contribution of genetic variation in taste receptors to food selection.

The putative fat taste receptor CD36, the sweet taste receptor T1R2, and the bitter taste receptor T2R38 contain common polymorphisms which may alter taste perception, food preferences, and consequently snacking patterns. Thus, the present study aimed to determine the relationship between SNPs in *CD36*, *TAS1R2*, and *TAS2R38* taste receptor genes and snacking patterns measured in children aged 1.5–5 years in the Guelph Family Health Study pilot.

## 2. Materials and Methods 

### 2.1. Study Design

To investigate the effect of genetic variation in taste receptors on snack consumption in preschoolers, baseline data from the Guelph Family Health Study (GFHS) pilot were analyzed cross-sectionally. The GFHS is a family-based cohort study that aims to understand early life risk factors for chronic disease and test ways to help families maintain healthy behaviours over many years. Families were eligible to participate if they had at least one child aged 1.5–5 years at the time of recruitment, lived in the Wellington County area, and did not plan to relocate outside of this area within the first year of the study. Forty-four families participated in the GFHS pilot study. Forty-seven preschoolers from 38 families attended a health assessment visit during which saliva was collected and body mass index (BMI) was measured. Preschoolers’ dietary intake from snacks was assessed using parent-completed three-day food records. All study procedures were administered after the parents of the participants gave written, informed consent. The study was approved by the University of Guelph Research Ethics Board (REB#14AP008).

### 2.2. SNP Genotyping

Saliva was collected using the Oragene·DNA (OG-575) collection kit for Assisted Collection (DNA Genotek). Participants were fasted for a minimum of 30 min before the saliva sample was provided. Genetic material from saliva was extracted by ethanol precipitation according to the manufacturer’s protocol (DNA Genotek). Real-time polymerase chain reaction (RT-PCR) was used to identify the genotypes of the participants with respect to the *CD36* (rs1761667; Assay ID C___8314999_10), *TAS1R2* (rs35874116; Assay ID C_____55646_20), and *TAS2R38* SNPs (rs713598; Assay ID C___8876467_10). Taqman fluorescent oligonucleotide probes were obtained for each SNP (Thermo Fisher Scientific, Waltham, MA, USA, Cat. #4351379). A BioRad CFX96 Touch™ RT-PCR Detection System in tandem with the Bio-Rad CFX Manager Software was used to detect the fluorescent signals and to produce a graphical representation which allowed for allelic discrimination. 

### 2.3. Snack Frequency, Quantity, Quality and Composition

Parents completed a three-day food record for their children, including two weekdays and one weekend day. Parents documented a detailed description of each food item (i.e., cooking method, brand name) and the amount of the food or beverage consumed. Food records were inputted into a nutrient analysis program (ESHA Food Processor, Version 11.0.110, Salem, OR, USA). ESHA contains a database of over 55,000 food items from over 1700 reputable sources including the Canadian Nutrient File. Using an average of the three-day food records, snacking data were analyzed based on quality (total energy density and nutrient composition), quantity (total energy intake), and frequency of snacks in the morning, afternoon, and evening. Foods and/or beverages (except water) consumed between meals were recorded as snacks, as previously described in the GFHS [33]. The average daily energy density of snacks (kcal/g) was calculated by dividing the total energy intake from snacks by the total weight of snacks. Percent caloric intake from fat, carbohydrates, and sugar was determined by dividing the calories from each nutrient component in snacks by the total calories of snacks. Quantity was determined as total energy from snacks (kcal/day). Frequency was calculated as the number of snacks consumed per day, and these were divided between morning, afternoon (between lunch and dinner), and evening snacks.

### 2.4. Data Analysis

Data were analyzed using SAS version 9.4 (©2012–2016, SAS Institute Inc., Cary, NC, USA). Generalized estimating equations were used to estimate the regression coefficients for linear models of snack measure outcomes and SNP predictors, with BMI *z*-score as a covariate. Frequency of evening snacking was normalized by a square transformation to obtain a normal distribution. All analyses account for correlated outcomes resulting from multiple siblings within some families. Results are presented as least squares means (LSM) and were not adjusted for multiple testing due to the exploratory nature of the study. Statistical significance was set at *p* < 0.05. Recessive, dominant, and additive models were used to analyze rs1761667 (AA vs. AG/GG), rs35874116 (CC/CT vs. TT), and rs713598 (GG vs. CG vs. CC) respectively in line with previous research [6,20,34].

## 3. Results

### 3.1. Participant Characteristics and Snacking Patterns

The mean age of the participants was 3.5 ± 1.2 years. Approximately half of the participants were male. The children were mostly of Caucasian origin and a wide range of family incomes was represented (Table 1). Snacking patterns show that all of the children snacked daily, with snacks constituting 32% of total daily energy intake (Table 2).

### 3.2. Association of SNPs at the CD36, T1R2, and T2R38 Genes with Snacking Patterns

**Figure 1:**
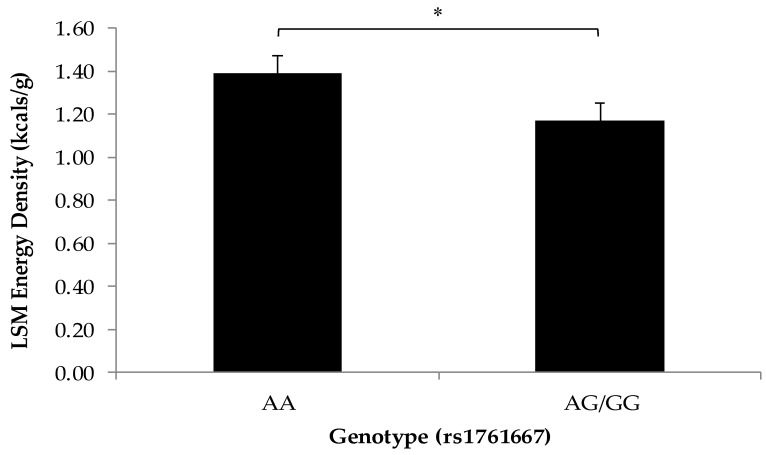
Least squares means of energy density of snacks by rs1761667 genotype Statistical differences between the total energy density of snacks by genotypes for rs1761667 of the *CD36* fat taste receptor gene were determined using generalized estimating equations (*n* = 47; * *p* = 0.007) and a recessive genetic model [6]. Children carrying the AA genotype (*n* = 13), predicted to prefer fat more than the AG/GG genotypes (*n* = 34), consumed snacks with a higher total energy density than children carrying the G allele. Values are reported as least squares means + SE.

There were no significant differences in BMI *z*-score among the participants based on any SNP genotype. The total energy density of snacks was greater in the AA genotype (1.39 ± 0.08 kcal/g) of the *CD36* SNP compared to G allele carriers (1.17 ± 0.03 kcal/g) (Figure 1). There were no significant differences in other snack measures by *CD36* genotype related to snack frequency, quantity, or calorie composition. 

**Figure 2:**
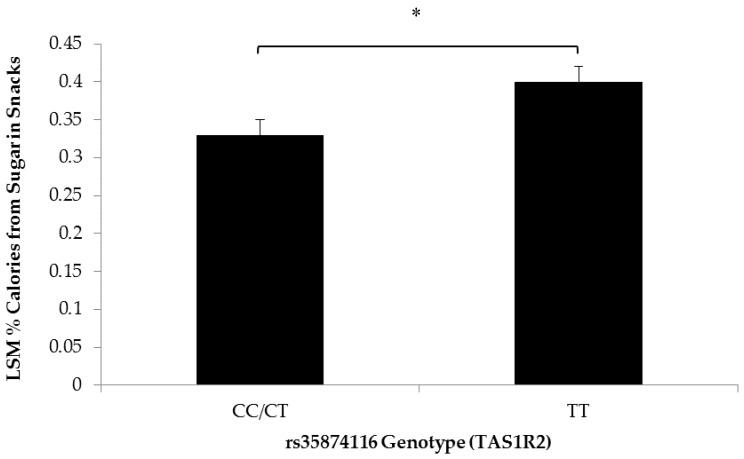
Least squares means of the percent calories from sugar in snacks by rs35874116 genotype Statistical differences between the relative calories from sugar in snacks for rs35874116 of the *TAS1R2* sweet taste receptor gene were determined using generalized estimating equations (*n* = 47; * *p* = 0.008) and a dominant genetic model [20]. Children carrying the TT genotype (*n* = 25), predicted to prefer sweet, consumed snacks with significantly more calories from sugar than carriers of the C allele (*n* = 22). Values are reported as least squares means + SE.

**Figure 3:**
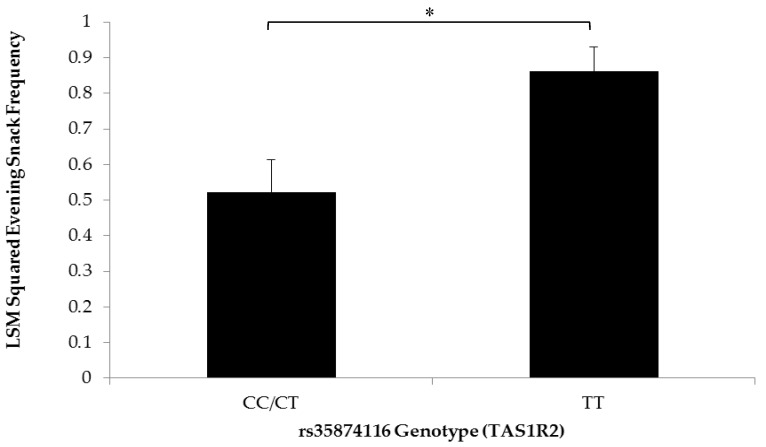
Least squares means of squared evening snack frequency by rs35874116 genotype Statistical differences between the square-transformed frequency of evening snacks for rs35874116 of the *TAS1R2* sweet taste receptor gene were determined using generalized estimating equations (*n* = 47; * *p* = 0.004) and a dominant genetic model [20]. Children carrying the TT genotype (*n* = 25), predicted to prefer sweet more than carriers of the C allele (*n* = 22), consumed more snacks in the evening. Values are reported as least squares means + SE.

Children with the TT genotype of the rs35874116 SNP in *TAS1R2* consumed snacks with higher % calories from sugar than children carrying the C allele (0.40 ± 0.02 and 0.33 ± 0.02, respectively) (Figure 2). In addition, the square-transformed frequency of evening snacks was higher (0.86 ± 0.07) for children with the TT genotype compared to C allele carriers (0.52 ± 0.09) (Figure 3). Based on a qualitative categorization of snacks consumed in the evening, the type of snacks consumed were also significantly more likely to be sugary snacks for children carrying the TT genotype (*p* = 0.04). However, there were no significant differences in measures of snack quantity and snack frequency in the morning and afternoon. 

**Figure 4:**
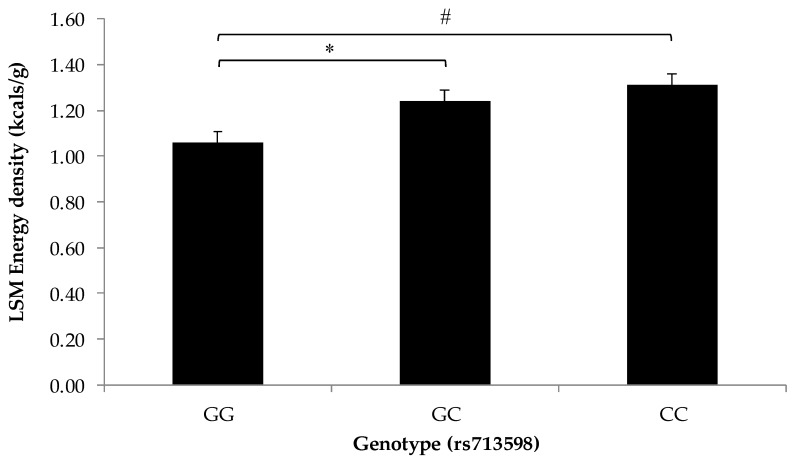
Least squares means of total energy density of snacks by rs713598 genotype Statistical differences between the total energy density of snacks by genotypes for rs713598 of the *TAS2R38* bitter taste receptor gene were determined using generalized estimating equations (*n* = 47; * *p* = 0.01, # *p* = 0.001) and an additive genetic model [34]. Children carrying the CC (*n* = 12) or CG (*n* = 27) genotypes consumed snacks with an overall higher energy density than the children carrying the GG genotype (*n* = 8). Values are reported as least squares means + SE.

The total energy density of snacks was highest in the CC genotype (1.31 ± 0.06 kcal/g) and CG genotype (1.24 ± 0.05 kcal/g) compared to GG genotype (1.06 ± 0.05 kcal/g) of the rs713598 SNP in *TAS2R38* (Figure 4). There were no differences in other snack measures by *TAS2R38* genotype related to snack frequency, quantity, or calorie composition.

## 4. Discussion

The present study showed that genetic variation in taste receptors are associated with potentially unhealthy snacking patterns in children. Three SNPs were investigated within taste receptor genes for fat (*CD36*; rs1761667), sweet (*TAS1R2*; rs35874116), and bitter taste (*TAS2R38*; rs713598). The prevalence of snacking in this cohort is similar to the prevalence of snacking in American children, and the calories from snacks are similar as well [31]. In addition, in a Brazilian study, the prevalence of snacking in children can be divided into children who are light-snackers and heavy-snackers. The prevalence of snacking and the calories from snacks are similar between heavy-snackers in Brazil and children from North American cohorts. However, the light-snackers in Brazil consume less snacks which also comprise a smaller portion of calories than North American children [35].

Adults with the A/A genotype of the rs1761667 variant have been shown to have lower oral sensitivity to fat and higher preference for fat [6,36]. In the present study, no relationship was observed between carrying the AA genotype and snack quantity, frequency, or calories from fat; however, children carrying the AA genotype consumed higher energy density of snacks than children carrying the G allele at this locus. While studies have shown differences in sensory outcomes based on this *CD36* SNP, an effect on dietary intake has never been observed. The present finding suggests that a fat taste receptor SNP genotype may influence the intake of energy-dense snacks. This finding is potentially due to the decreased sensitivity to fat taste and subsequent higher preference for fatty foods; however, measures of taste sensitivity were not determined in this study. Although the relationship between fat taste sensitivity and energy density of snacks in this study is consistent with the energy density of fat as a macronutrient, no difference was found between the calories from fat in snacks for this genotype. This apparent contradiction may be due to the challenges of measuring the contribution of fat in a complex diet which contains foods simultaneously high in fat and carbohydrates. Future research studies would benefit from performing hedonic tests for fat taste with children in more controlled settings in addition to tracking food records. A genetic predisposition to prefer fatty foods due to lower taste sensitivity may be of concern for children in the long term as high fat diets may lead to metabolic complications such as obesity, MetS, and CVD [37]. 

Carriers of the TT genotype at the rs35874116 locus (Ile191Val) in the *TAS1R2* sweet taste receptor gene have been shown to prefer sweet foods [20]. Children in the GFHS cohort carrying the TT genotype were found to consume snacks with significantly more calories from sugar than children carrying the C allele. While the percent of calories from sugar was different, it is not clear why the energy density of snacks was not significantly different based on rs35874116 SNP genotype. As snacks contain a complex combination of different macronutrients, it is difficult to discern which macronutrients contribute a higher proportion of energy density. While consistent with previous research linking the Ile191Val variant with habitual sugar consumption in overweight/obese adults and dental caries in children [20,23], the present finding demonstrates that taste preferences and dietary intake may also be related in children. Upon further analysis, it was found that sugary snacks were most frequently consumed in the evening by children with the TT genotype. If the evening is a time of day when children with a “sweet tooth” potentially consume more sugary snacks, this finding warrants future studies to assess the availability and accessibility of sugary foods in the home environment as a means to address the role of parents in the intake of sugary foods by young children during the evening. Future studies may also benefit from including genetic variants in the *TAS1R3* sweet taste receptor gene to examine determinants of sweet food intake [21,38]. Chronic overconsumption of sugar is a well-known risk factor for MetS and T2D [39]. With the advancement of research pertaining to this variant, there may be reason to use genotype at this locus as a risk factor for overconsumption of sugar in children. Adapting the Ile191Val SNP genotype into a clinical biomarker of dietary intake patterns in children may be helpful to mitigate the risk of developing obesity, MetS, and T2D.

The bitterness of green leafy vegetables (Brassica vegetables), related to the taste of thiol compounds, may be stronger in those homozygous for the C allele at the rs713598 locus in the *TAS2R38* taste receptor gene. Those who do not carry the C allele may not taste PTC, and this may then influence the perceived bitterness of Brassica vegetables [30]. We hypothesized that carriers of the CC genotype in our study would be more likely to consume energy-dense snacks due to their potential avoidance of bitter vegetables [4,40]. In support of this hypothesis, CC genotype carriers and CG genotype carriers had higher total energy density of snacks compared to GG genotype carriers. No relationships were observed between rs713598 genotype and snack quantity, frequency, or calorie composition, in line with previous research [41,42]. This finding contributes to a growing, controversial body of literature about this genetic locus, showing that this genotype may adversely affect quality of dietary intake [43]. Similar to the other variants investigated in the present study, this SNP may serve as a biomarker for at-risk eating patterns with the potential of developing into adverse metabolic and health outcomes.

The prevalence of combinations of the taste receptor SNP genotypes associated with snacking patterns are important to consider. Participants can be classified by the proportion of “at-risk” genotypes they carry based on the three SNPs included in the present analysis. The “at-risk” genotypes were AA for the *CD36* SNP (fat preference), TT for the *TAS1R2* SNP (sweet preference), and CC for the *TAS2R38* SNP. None of the children in this study had all three “at-risk” genotypes, 13/47 (28%) of the children had two “at-risk” genotypes, 24/47 (51%) of the children had one “at-risk” genotype, and 10/47 (21%) of the children had no “at-risk” genotypes. Therefore, the prevalence of carrying at least one “at-risk” genotype was almost 80% within this cohort. This observation is an indicator that these genotypes are sufficiently common and warrant parents’ awareness of the potential influence on habitual dietary intake in young children. It is not known whether the potential disadvantage of carrying “at-risk” genotypes is additive when a combination of these genotypes is present. Moreover, it is difficult to assess whether one “at-risk” genotype can be cancelled out by a “healthy” genotype at a different locus within the same taste receptor gene. Analysis of the relative influences that each SNP has on habitual dietary intake requires a greater understanding of the individual effect on habitual dietary intake as well as a much larger study cohort. 

This study’s limitations should be considered when interpreting the results. These data were obtained in a pilot phase of the GFHS, and the probability of type 2 error is higher with a small sample size. Due to the exploratory nature of this study, multiple testing was not accounted for and there is a possibility of false discovery in the findings. Furthermore, there is uncertainty that the quantity, quality, frequency, or composition of snacks consumed reflect the taste preferences of the children rather than parental feeding styles and practices. While it is probable that taste preferences are strong indicators of food selection in this study, the parents’ influence on food availability and accessibility need to be considered in future investigations.

## 5. Conclusions

Overall, this study suggests that genetic variation in taste receptors for fat, sweet, and bitter taste may be associated with snacking patterns in young children. The extent to which the SNPs affect dietary intake must be investigated in future studies by elucidating the mechanisms with which the SNPs alter taste receptor function, and how snacking patterns are subsequently influenced by changes in taste perception.

## Figures and Tables

**Table 1:** Participant Characteristics.

**Sex**	
Male	*n* = 22, 47%
Female	*n* = 25, 53%
Age (years, mean (SD))	3.47 (1.15)
BMI *Z*-Score (mean (SD))	0.43 (0.99)
**Ethnicity (%)**	
Caucasian	87.5
Other	12.5
Parent annual household income (Canadian $), *n* = 37 * (%)	
≤$49,999	18.9
$50,000–$79,999	21.6
$80,000–$99,999	21.6
≥$100,000	37.8

* Total families that provided parent annual household income (missing data, *n* = 1).

**Table 2:** Participant Snacking Patterns.

Snacking Measure	Mean (SD) (*n* = 47)
Total daily energy intake (kcals/day)	1407 (347)
Total energy intake from snacks (kcals/day)	456 (213)
% Daily energy from snacking	32 (14)
Total energy density of snacks (kcals/g)	1.13 (0.43)
Total snack consumption frequency (%)	78 (39) *
Morning (%)	100 (33) *
Afternoon (%)	100 (33) *
Evening (%)	67 (67) *

Overall participant snacking patterns were computed (*n* = 47). * Variable was not normally distributed: median and interquartile range (IQR) used as measures of center and spread.
